# Web search behaviors and infodemic attitudes regarding COVID-19 in Turkey: A framework study for improving response and informing policy on the COVID-19 infodemic

**DOI:** 10.3389/fpubh.2022.948478

**Published:** 2022-11-08

**Authors:** Cüneyt Çalışkan, Gözde Özsezer, Melek Pay, Gülcan Demir, Ismet Çelebi, Hüseyin Koçak

**Affiliations:** ^1^Department of Emergency Aid and Disaster Management, Hamidiye Faculty of Health Sciences, University of Health Sciences, Istanbul, Turkey; ^2^Department of Public Health Nursing, Nursing Faculty, Ege University, İzmir, Turkey; ^3^Department of Paramedic, Fethiye Vocational School of Health Services, Muǧla Sıtkı Koçman University, Muǧla, Turkey; ^4^Vocational School of Health Services, Sinop University, Sinop, Turkey; ^5^Department of Paramedic, Gazi University, Ankara, Turkey; ^6^Department of Emergency Aid and Disaster Management, Faculty of Health Sciences, Çanakkale Onsekiz Mart University, Çanakkale, Turkey

**Keywords:** COVID-19, disasters, pandemics, infodemic, web searches, Maslow's hierarchy of needs, fears COVID-19-related

## Abstract

**Objective:**

This study aimed to develop a framework regarding COVID-19 infodemic response and policy informing through focusing on infodemic concepts circulating on the online search engine in Turkey in relation to the COVID-19 outbreak and comparing the contents of these concepts with Maslow's hierarchy of needs and disaster stages.

**Materials and methods:**

The universe of this descriptive epidemiological research consists of internet search activities on COVID-19 circulating online on Google Trends between March 10, 2020, when the first case was seen in Turkey, and June 01, 2020, when the lockdown restrictions were lifted.

**Findings:**

There was no internet trend regarding a misinformed attitude within the given date range. While an infodemic attitude toward superficial attitude and racist attitude in the internet environment was detected for 1 week, an infodemic attitude toward definitive attitude was detected for 2 weeks. The non-infodemic concepts were more common than the other infodemic attitudes. The infodemic concepts were able to reach Maslow's physiological, safety, and social need levels. With the infodemic concepts obtained, a COVID-19 development process framework was developed. The framework consists of three domains (COVID-19, applications and outcomes), including disaster phases and health/social impacts, built on seven public health epochs.

**Results:**

A systematized COVID-19 development process framework was modeled in order to conceptualize COVID-19 internet searches and to reveal the development processes and outcomes.

## Introduction

The COVID-19 pandemic poses unprecedented challenges for society and health systems worldwide. As of the end of July 2021, the World Health Organization (WHO) reports over 194 million confirmed cases globally, more than 4.1 million deaths, and more than 1.4 billion at least one dose of vaccine administration. In Turkey, there are more than 5.6 million confirmed cases, more than 50 thousand deaths, and more than 38.9 million at least one dose of vaccine application (1). In the fight against the pandemic, like many countries (2), Turkey tries to prevent and eliminate the waves of infection with a series of public health interventions such as travel restrictions, physical distance, and use of masks.

For the first time in history, the COVID-19 pandemic has occurred at a time when technological developments and social media are frequently used to keep people safe, informed, productive and connected. Many countries experience an infodemic problem that jeopardizes humanity's measures to contain the pandemic globally (3). According to the World Health Organization, infodemic is defined as such: An infodemic is too much information including false or misleading information in digital and physical environments during a disease outbreak (4). According to Cambridge Dictionary, misinformation refers to “(1) wrong information, or the fact that people are misformed, and (2) information intended to deceive, whereas disinformation means “false information spread in order to deceive people” (5). There is an intent difference between misinformation and disinformation. The concept of disinformation is used in cases where there is malicious intent or an intention to cause harm in the dissemination of information (6).

An infodemic involves attempts to deliberately disseminate misinformation to undermine people's compliance with public health practices and to develop an alternative agenda. Misinformation and disinformation harm people's physical and mental health, putting countries' efforts to combat the pandemic. In the short term, this could endanger various public health efforts, such as vaccination campaigns; similarly, in the long run, it can also lead to polarization of public debates about the pandemic, to a conflict among marginalized groups, and to human rights violations (3). In particular, as reported by Dr. Tedros Adhanom Ghebreyesus from WHO, not only is a pandemic being fought, but also an infodemic. The transmission, repetition and continuation of mistakes and conspiracies through social media and traditional methods make public health studies disadvantageous. In this period of uncertainty, there is a need to rapidly disseminate reliable information and to prevent societal panic disease with verified information (7).

The inability of countries to control the infodemic that occurs during the pandemic drives them to various mandatory practices such as introversion and isolation. In addition to increasing epidemiological studies on the virus and the process of controlling the disease, it is seen that it is necessary to participate in other disciplines that intersect with the relevant subject (8). In this context, it may be beneficial to review some of the social consequences of the pandemic on society. For instance, decision makers can urgently incorporate Maslow's hierarchy of needs into their policies to resolve virus-related crises. Because Maslow's hierarchy of needs provides a framework for understanding the effects of the system on society and what motivates people. In the hierarchy of needs, from the lowest level to the top (physiological needs, safety needs, love and belonging, esteem, and self-actualization, respectively), there is the desire for the fulfillment of human expectations (9). In this respect, it can be said that identifying and understanding human needs (10) can provide an accurate definition of pandemic response and recovery strategies. In particular, with the identification of subjective concerns (11) or needs, expected human behaviors can be structured in a desired way (10). In this context, since the pandemic process is a phenomenon that affects societies, our longitudinal (before and after) understanding of disaster risk management with Maslow's theory should be developed. In order to carry out disaster risks in a systematic framework, there is a need for improvement studies in order to define risks before the disaster and implement preparedness studies, to implement pre-determined response policies during the disaster, and to build the old one better in the last stage after the disaster.

The United Nations Secretary-General launched the UN Communications Intervention Initiative in April 2020 to combat the spread of misinformation and disinformation. In May 2020, the World Health Assembly decided that managing the infodemic was a critical part of controlling the COVID-19 outbreak. WHO called on its member states to provide standard COVID-19 content, to take measures against misinformation and disinformation, and to take advantage of digital technologies (3). In this direction, (1) the detection of infodemic concepts circulating in the online search engine in Turkey, (2) the discussion of these contents in terms of Maslow's hierarchy of needs and disaster stages, and (3) It is aimed to develop a framework that presents the development process of COVID-19 to improve response and inform politicians regarding the COVID-19 infodemic.

## Materials and methods

The universe of this descriptive epidemiological research consists of internet searches related to COVID-19 circulating online on Google Trends between 10 March 2020, when the first case was seen in Turkey, and 01 June 2020, when the lockdown restrictions were lifted. In the research, interpretation errors, fake news, racism incidents and other misleading information circulating on the internet were defined as COVID-19 infodemic concepts.

### Demographic structure

Turkey has a population of over 84 million as of 2021, and 50.1% of the population is men. The median age of the population is 33.1 and 67.9% of the population is between 15 and 64 years old, which is defined as working age population (12). The rate of internet usage in the country was measured as 82.6% in 2021 for individuals in the 16–74 age group (13).

### Data extraction and classification

Only Google Trends was used as the data source in the study because Google Trends is the most popular tool for addressing health problems and issues with the use of internet data. Google is an online tool that provides both real-time and archived information, and Google Trends can extract information from people who identify or disguise themselves in real time that is difficult and impossible to collect. Trends is an online tool, which tracks keyword search queries that users enter on the Google search engine, and determines their popularity and volume. It also provides information about the search query based on a specific time period and location. Search volume results scale from 0 (very low) to 100 (very high) (14). Data extraction from Google trends was conducted on February 15, 2021. The data were analyzed in 12 week time periods, including the following date ranges: 10–15 March 2020, 16–22 March 2020, 23-29 March 2020, 30 March- 5 April 2020, 6–12 April 2020, 13-19 April 2020, 20–26 April 2020, 27 April-3 May 2020, 4–10 May 2020, 11–17 May 2020, 18–24 May 2020, and 25 May-1 June 2020. When Turkey and the above-given date ranges were selected together from the Explore tab of Google Trends, a list of Google web searches for the country was obtained. In the results section of the list, there are “search subject” and “search query” sections. In these sections, prominent concepts related to COVID-19, which are in the top 20 in the list given by Trends, were drawn.

### Analysis

The frequencies of the search words obtained from Google Trends were given by matching the infodemic concepts and Maslow's hierarchy of needs.

#### Sentiment analysis

In the study, the general sentiment analysis method, which provides the assignment of a single emotion to a content, was used. Here, the semantic or content meaning of the words used in web searches was searched or matched. The COVID-19 concepts identified in the above-defined date range were matched with the infodemic, Maslow's hierarchy of needs, and disaster phase sub-themes defined below. Integration of concepts with a theme was accepted as a criterion in matching. The validity of the matchings was conducted by the two researchers of the study:

- *Matching data with infodemic groups:* The lists described above were divided into 4 groups within the scope of infodemic data, as in the study of Rovetta and Bhagavathula (15): (1) Superficial attitude: The user adopts words which can cause confusion (e.g., coronavirus) as the subject uniquely describes it; (2) Misinformative attitude: The user adopts words which can lead to the spread of fake news (e.g., 5G coronavirus); (3) Racist attitude: The user willingly or unintentionally adopts words which create or emphasize incidents of racism (e.g., Chinese coronavirus); (4) Definitive attitude: The user adopts the most appropriate conditions for the correct identification of the query (e.g.,: COVID-19); (5) Non-infodemic queries: The user investigates other information about the pandemic (e.g., paper mask making).
- *Matching data with Maslow's hierarchy of needs:* The concepts obtained through Google Trends provide a framework for understanding the impact of COVID-19 on society and what motivates them. In this direction, a relationship was established between the concepts and Maslow's hierarchy of needs, and a matching was carried out. Maslow's hierarchy of needs (16) consists of the following needs: (1) physiological needs such as hunger, thirst, sleep and shelter; (2) security needs such as employment, access to resources, property, and health; (3) social needs such as belonging, love, acceptance and social life; (4) esteem needs such as status, achievement, recognition and recognition; (5) self-fulfillment needs such as development, accomplishing a job successfully, and creativity.
- *Matching COVID-19 development process framework through disaster phase:* Disaster phases consist of the following phases: (1) hazard prevention or risk mitigation and prevention, (2) preparedness efforts to mitigate the negative consequences of disaster, (3) intervention to reduce damage and losses in disasters, and (4) post-disaster restructuring improvement/rehabilitation phases (17).

#### Theoretical framework of COVID-19 development process

Inspired by the health stigma and discrimination framework study of Stangl et al. (18), a holistic perspective was put forward by bringing together the data obtained from Google Trends on Maslow's hierarchy of needs, disaster phases, and public health epochs. This perspective was based on the developmental stages of public health in a historical process, and the COVID-19 pandemic process was consequently examined. The development process of the pandemic was divided into three domains:

- In the COVID-19 domain, the first domain refers to the factors that facilitate or direct the spread of the virus in the community. Similarly, drivers cover the social determinants of health focusing on a wide range of health themes in environments where people are born, live, learn, work, play, worship, and get older (19). The domain of facilitators has both positive and negative contributions to behavior. Drivers and facilitators systematize the dynamics that define the spread of COVID-19 in the society. Maslow's hierarchy of needs is the reference point that defines the place of a society in this structure and can guide the policies of decision makers. At this point, with sentiment analysis, it was revealed which domain of need data corresponded to.
- Applications–the second domain–refer to the social and institutional arrangements and practices carried out by the government for the prevention of the spread of COVID-19 in the society. These practices are divided into two, both at the individual and social level, and at the organizational and institutional level. Such applications are carried out as an intervention to the findings obtained from the domain of COVID-19.
- The third domain, outcomes, refers to the final outcomes of the actions described in the previous domains to prevent the spread of COVID-19 in the community. In this domain, the results of all actions taken to prevent the disease can be seen at the individual and social level, as well as at the organizational and institutional level.

The health and social impacts of these three domains are shown at the top of the figure. These are defined as disease-specific incidence, morbidity, mortality, and quality of life. On the left side of the figure, measures taken against the pandemic and applications are defined specifically for disaster phases.

### Ethical approval

Ethical approval was not required as all materials were derived from anonymous open source data.

## Findings

There are 12 weeks between March 10, 2021, when the first case was observed in Turkey, and June 1, 2021, when the first lockdown restrictions were lifted. An internet trend regarding a misinformed attitude was not recorded in any of these weeks. While an infodemic attitude toward superficial attitude and racist attitude in the internet environment was detected for one week, an infodemic attitude toward definitive attitude was detected for 2 weeks. The non-infodemic concepts were more common than the other infodemic attitudes (Table 1). Other concepts associated with COVID-19 were able to reach Maslow's physiological, safety, and social need levels (Table 2).

**Table 1:** Distribution of infodemic concepts related to COVID-19.

**Week**	**Infodemic group 1–Superficial attitude**	**Infodemic group 2–Misinformative attitude**	**Infodemic group 3–Racist attitude**	**Infodemic group 4–Definitive attitude**	**Non-infodemic queries 5**
1	Corona symptoms				Cologne, Pandemic, Sanitizer, Mask
2				Favipiravir COVID-19	Fahrettin Koca
3			Hantavirus	COVID-19	Intubation
4					Mask, Intubation
5					Mask
6					Mask, Life Fits into Home, Ercüment Ovali
7, 8, 9, 10					
11					PTT shipping (mask distribution)
12					
Total	1 week	0 week	1 week	2 weeks	7 weeks

**Table 2:** Matching other concepts related to COVID-19 with Maslow's hierarchy of needs.

**Weeks**	**Maslow**	**Frequency**
1	Are schools on holiday = Social	1
2	Lockdown = Security Eba TV = Social Bread = Physiological	3
3	Sumac = Physiological Job application = Security Zoom = Social Eba TV = Social Bread recipe at home = Physiological Bread = Physiological	7
4	TV series = Social Loan application = Security Zoom = Social The Bizbizeyeteriz campaign = Security	5
5	The Açik Kapi support application = Security Loan application = Security Lockdown = Security Bakery = Physiological	4
6	Loan application = Security When will schools open = Social	2
7	Pandemic and social service = Social	1
8	Lockdown = Security Life Fits into Home = Social Loan application = Security Social assistance application = Security Eba TV = Social	5
9	Bill on the Hook = Security Loan application = Security Pandemic = Security	3
10	Game = Social Travel = Social Lockdown = Security Loan application = Security Pandemic = Security	5
11	HES code = Security Market = Physiological Lockdown = Security	3
12	Lockdown = Security Coronavirus table = Security Normalization calendar = Security Loan application = Security	4
Total		43

**Figure 1:**
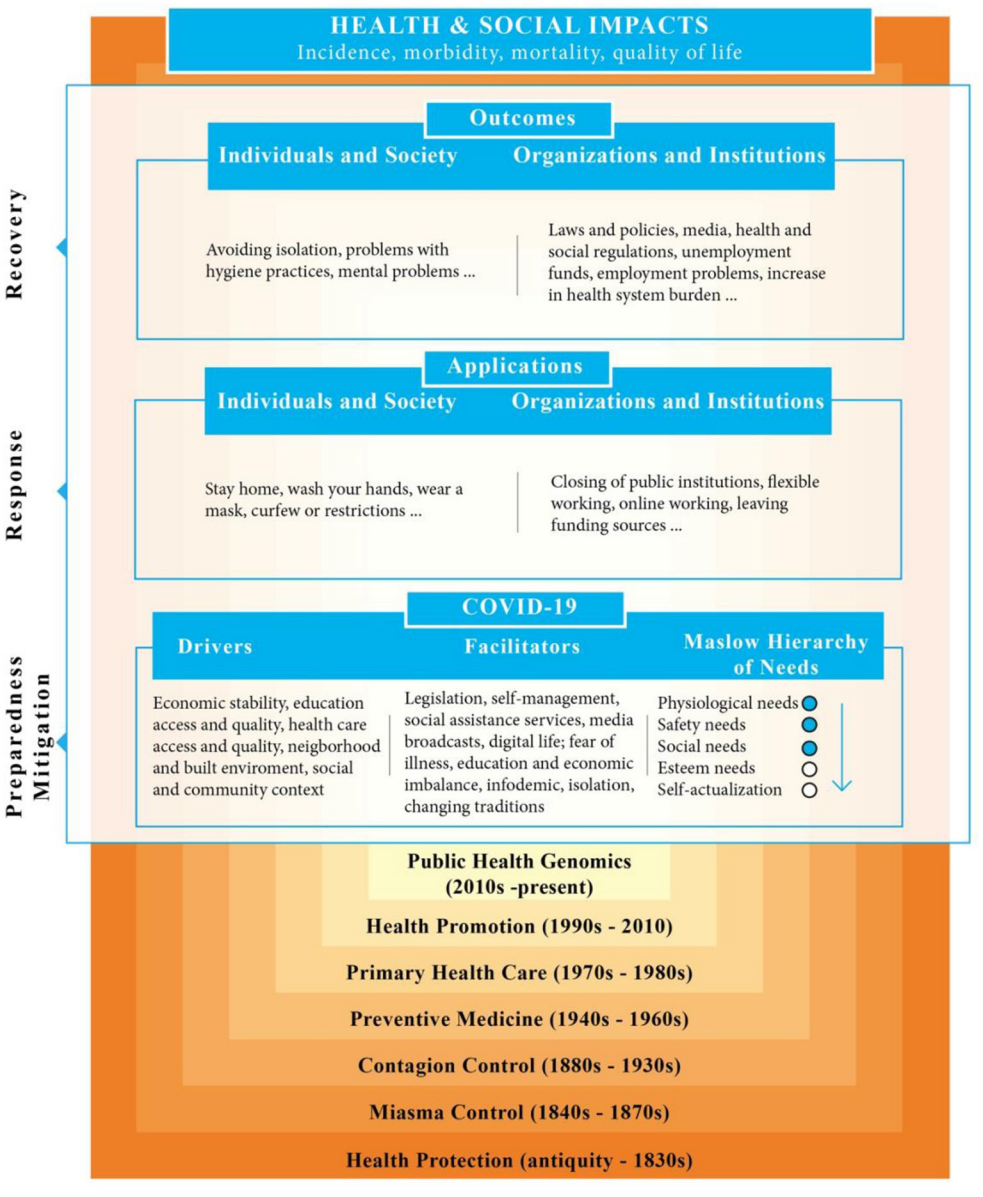
COVID-19 development process framework.

The COVID-19 development process framework consists of three domains, disaster stages, and health/social impacts, built on seven public health epochs. The COVID-19 drivers in Figure 1 consist of economic stability, education access and quality, health care access and quality, neighborhood and built environment, and social and community context, whereas the facilitators are legislation, self-management, social assistance services, media broadcasts, digital life, fear of illness, education and economic imbalance, infodemic, isolation, and changing traditions (Figure 1).

The applications in Figure 1 consist of individual and social structures as well as organizational and institutional structures. While individual and social applications include stay home, wash your hands, wear a mask, and curfew or restrictions, organizational and institutional applications include closing of public institutions, flexible working, online working, and leaving funding sources (Figure 1).

The outcomes domain in Figure 1 refers to the final outcomes of the actions, which were defined in the previous domains to prevent the spread of COVID-19 in the community, under two groups. Individual and social outcomes include avoiding isolation, problems with hygiene practices, and mental problems. On the other hand, among the organizational and institutional outcomes are laws and policies, media, health and social regulations, unemployment funds, employment problems, and increase in health system burden (Figure 1).

The health and social effects of the COVID-19, applications and outcomes domains in Figure 1 are shown at the top of the figure. These are defined as disease-specific incidence, morbidity, mortality, and quality of life. On the left side of the figure, disaster management's mitigation, preparation, response and recovery phases are referred to, which facilitates the management of an event by dividing it into phases and transforming it into a systematic form. Here, activities are carried out on minimizing the effects of a disaster by mitigation, planning the scope of preparedness and response, minimizing and rehabilitating the hazards posed by a disaster with response, and returning to normal life (Figure 1).

## Discussion

In the study, discovering the infodemic concepts circulating online in Turkey, comparing them with Maslow's hierarchy of needs, and disaster phases, and discussing them within the context of public health epochs were aimed. In this direction, a COVID-19 development process framework was developed.

### Infodemic

Tedros Adhnom Ghebreyesus, the general manager of WHO, used the phrase “We're not just fighting a pandemic; we're fighting an infodemic” at the 2020 Munich Security Conference. This sentence emphasizes that not only a disease that threatens the health of the world should be tackled, but also the confidence of countries in health systems and programs should be kept alive (20). In this struggle, Sylvie Briand, the architect of WHO's strategy to counter the infodemic, states that misuse of social media spreads false information rapidly (21). In some studies conducted in this context, according to a confirmed information, it was shown that fake news and false information spread in a wider area and faster on social media (22, 23). From this point of view, WHO continuously provides up-to-date information through social media and website, encouraging people to act in the right way. In a similar way, within the scope of combating the infodemic, Turkey also started to work on print and visual media shortly after the virus appeared, taking WHO as a reference (24).

In line with the findings obtained in the study, it was determined that the society did not have a superficial attitude that could cause confusion regarding the COVID-19 infodemic and that they did not have a misinformed attitude which could cause the spread of fake news. As in the rest of the world, Turkey goes through a pandemic period in which fake news, false information, conspiracy theories and scientific data presented to the public without comprehensive examination are shared. This chaotic period provides an environment where researchers constantly learn and where these experiences are applied dynamically by politicians (20). Especially in the fight against fake news, expanding health (22, 25) literacy, establishing web-based guide sites, imposing prison sentences, cooperating with social media companies to close some accounts, and using artificial intelligence algorithms in tracking fake news are among the options (22). On the other hand, algorithms enable the promotion of content and facilitate the dissemination of information through the consideration of account users' preferences and attitudes (26). For this reason, online participants, who tend to obtain information from sources close to their own worldviews, ignore opposing information and polarize around shared narratives, which causes the spread of true or false information (26, 27). In fact, such environments constitute the center of an infodemic war (27). From this point of view, although there are various tools in the fight against an infodemic, its spreading sources are also the means of struggle.

It was determined that users, willingly or unwillingly, used a racist discourse in their web search queries. It can be said that this concept is not a racist statement, but an interrogation related to the region where the virus first appeared. Similar examples have been seen in the cities of Italy (15) or in the trending hashtag # ChineseDon'tComeToJapan on Japanese Twitter. There are also rumors that Chinese restaurants are losing jobs in the USA and that France is dealing with an anti-Asian racism pandemic (28). These examples can be further reproduced by examining other countries. It constitutes a key component in the development of racism and xenophobia, especially as fear is at the root of pandemics. The disease has caused social and political breakdowns in societies (29), leading to the marginalization of the Chinese (29, 30). In a period when the internet is removing borders, the Chinese virus rhetoric of Donald Trump, the former US President, or the desire of Matteo Salvini, Italy's former Deputy Prime Minister, to close the borders by accidentally linking the disease to African asylum seekers (29) is being watched by world citizens and is encouraging societies to racist actions. For this reason, politicians should pay attention to their discourse, and mass media should be used to provide and disseminate correct information among citizens.

Among the user queries, there are expressions which provide correct definition and contain precise attitude. Correct spelling of the disease name and researching the drug name used in treatment are the good examples of combating disinformation that multiplies and expands in an environment of uncertainty. Especially having accurate and timely information, citizen management and compliance, good governance, strict regulation, and proper use of big data and digital technologies can be among the important factors in stopping the spread of the disease (21). In addition to these factors, the world will continue to be a living scene of disinformation engineering until the vaccine application is completed within the scope of the final solution (31).

It was found that non-infodemic queries about the pandemic took place in a wide range of web searches. For example, researching a simple way to make a mask at home is a proof that people strive to protect their health. In this respect, public health education is important in the strategy to combat the disease (25). Again, apart from education issues, public health authorities and private technology companies can act together on contact tracking, pandemic modeling and the management of infodemic information in public health communication. However, in the fight against COVID-19, the threat of individual privacy by digital applications, increased government surveillance and control, and digital dependence on companies are a huge concern (32).

### Framework

COVID-19 has made public health studies more visible than ever in Turkey and enabled more resources to be transferred to public health practices. On the other hand, it has created a new classification of life characterized by physical distance, compulsory isolation, personal protection equipment, contact tracking, and material and moral assistance for many people (33). Although the new classification is intended to protect people, it creates negative feelings in most of them. It is thought that Maslow's human needs theory is very compatible with the negative emotions that arise within the context of COVID-19. According to Maslow's theory, human needs are hierarchically organized, and when a certain need is met, another arises and progresses in this way. This study, in this regard, refers to the first three levels of the Maslow pyramid and consequently shows the existence of connections between the perception of negative basic emotions caused by COVID-19 and meeting needs. As a result of the worldwide COVID-19 outbreak, many people have had to retreat to the lower levels of the pyramid, regardless of their location in the Maslow pyramid before the pandemic. This unexpected change in individual basic needs has triggered a transformation in the individual's emotional state and led to a shift toward negative emotions. The pandemic process has created an environment where people live in fear of losing the basic need level that they reached in Maslow's hierarchy of needs before the pandemic (34). Also, results from a general population can be even more devastating when supplemented with specific groups. For example, the importance of Maslow's hierarchy of needs for safety comes to the fore more in the applications made to psychiatric emergency services fighting on the front line regarding COVID-19 and in the studies exploring (35) the psychological effects of people living (36) alone or having a sense of loneliness and fear of contracting the disease. On the contrary, there are fears due to various reasons among health professionals who provide health services (37). This process involving all segments of society was detailed in the framework of the COVID-19 development process in Figure 1.

The COVID-19 development process framework refers to the COVID-19 process in the socio-ecological spectrum of health coverage in the context of countries with different economic levels. The process has been considered within the scope of outcomes which are built on the public health evolution of humanity (38–41) and ultimately affect health and societies. In addition, drivers, facilitators and applications, which affect the spread of COVID-19 among individuals and populations, have been explored in a number of areas within the scope of Maslow's hierarchy of needs and disaster phases. On the contrary, the lack of such a framework can be likened to decision-makers' efforts to determine when it is safe to ease constraints, what relaxation means, and whether such actions are helpful or harmful in the medium and long term (9).

The COVID-19 development process framework, designed to examine all the processes of a pandemic, basically rises above the public health evolution of humanity. Each evolution includes a new development and a new conservation approach within its body. This also causes a legitimacy crisis (41) of the method chosen in the context of modern health promotion. The best proof related to the fact that this will never end is that the seven epochs of public health have passed to date. The current epoch is expressed as public health genomics (38, 39). Public Health genomics studies focus on personalized medical studies in line with the specific genetic, physiological and psychological characteristics of each individual (40). The COVID-19 virus, on the other hand, has a mass impact as it turns into a pandemic. For this reason, all preventive and protective work carried out today is built on the achievements of the past. At the same time, the emerging framework reveals that personalized medicine may be needed in the treatment of new viruses that may emerge in the future, as well as gains from other epochs.

The COVID-19 domain in Figure 1 refers to the factors that facilitate or direct the spread of the virus in the community, as indicated in the findings. Drivers, on the other hand, cover the social determinants of health (19). These are expected to contribute positively to the prevention of transmission in societies with high economic and educational levels. However, since the development levels of the countries are different, it is possible that they will contribute negatively. Again, the facilitators section in Figure 1 has both positive and negative contributions. For example, while situations, such as regulations regarding COVID-19 disease and the responsibility perceived by the individual, may minimize the spread of the disease, cases such as fear of disease, education and economic imbalance may exacerbate the disease. However, in the study, it was not seen that cases such as economic instability increased the infodemic in internet searches. Drivers and facilitators systematize the dynamics which define the spread of COVID-19 in society. In other words, drivers and facilitators refer to the concept of “interpersonal theory of suicide” and “interpersonal trust” (42). It gathers “interpersonal theory of suicide” and insecurity caused by various stressors or dynamics, such as loneliness, unemployment and loss of income caused by COVID-19, in a single framework. Conversely, the concept of “interpersonal trust” protects mental and physical health by ensuring that dynamics that motorize the spread of COVID-19 are not intentional or intentional and that people are willing to accept the risk of illness. However, the reference point that defines the place of a society in these structures and can guide the policies of decision makers can pass through Maslow's hierarchy of needs (9). The pyramid can play a guiding role in the interventions to be implemented by defining the needs of a public at the local, regional and national levels. In line with the data obtained, it was determined that although the physiological and safety needs of the Turkish society were higher, they could reach the level of social needs. However, due to the design of the study, it is not known whether the need for security originates from government agencies. For that reason, there is a need to carry out observational studies with examples (43–45) in the literature. Economic instability, especially after COVID-19, has caused many people to lose access to employment. These people, who suffer from job loss now, will struggle with the stress of the job seeking process in the future (46). All of these can lead to increased social damage and instability and to the destruction of unmet social demands after a while if decision makers do not take measures in their current policies or do not develop interpersonal trust (42, 43).

Applications, the second domain in Figure 1, refers to the social and institutional arrangements and applications carried out to prevent the spread of COVID-19 in the community, as defined in the findings. It defines individual and social applications restricting people's mobility and compliance with certain hygiene rules. Organizational and institutional applications are based on methods based on working from home instead of traditional working procedures and deal with actions such as financial arrangements to meet the needs of the society. Applications may vary according to the dynamics of different countries, and regulations made in the fields of isolation at home, hygiene, education, health and finance constitute the main issues. Maslow's hierarchy of needs is not included in the applications domain of the framework, as in the COVID-19 domain. The level of applications can be determined by adding the hierarchy of needs to other future studies by researchers. In fact, at the first level, it can be determined how much the needs can be met with the applications put forward by the state in line with the needs or findings obtained from the searches of the society. This can be accomplished with an extension of the working method and a metadata analysis. In addition, another issue is that applications are fed and reinforced by drivers and facilitators at the first level. For this reason, the reflections of the combination of the social determinants of health and COVID-19 perception defined at the first level are seen in the applications domain.

The third domain in Figure 1 makes reference to the final outcomes of the actions described in the previous domains to prevent the spread of COVID-19 in society. Avoiding restrictions between individual and social outcomes, problems in complying with hygiene rules, and mental problems brought about by isolation at home are just some examples. Among the organizational and institutional outcomes, there are problems such as continuous legislation/regulation updates and the publication of new ones to ensure public order, access to education, employment problems, and burden on the health system. In this domain, the results of all studies to prevent the disease can be seen. Although the studies conducted are correct and accepted by the society, it can be said that negative outcomes will be higher until a definitive treatment is found. In addition, the link between the public needs level and outcomes identified in the first domain can be investigated here. This link can enable decision-makers to use scarce resources more effectively and efficiently in their policies and applications. This also means that an arrangement can be made by adding the hierarchy of needs here as well as in the domain of applications and in the domain of outcomes.

On the left side of the frame in Figure 1, a match was conducted with disaster phases. Although mitigation and preparedness are the processes carried out before a disaster occurs, they are matched with the first domain of the relevant framework. In fact, this may be an opportunity to restructure mitigation and preparedness in line with the first findings obtained from the disease. In addition, the rules within the scope of the regulations regarding the pandemic are among the good examples that can be given in relation to mitigation and preparedness. Apart from these, the treatment of the disease, restrictions, physical distance, release of various funds or new educational applications are evaluated within the scope of intervention or the second domain. The activities carried out within the scope of normalization of life with the start of relaxation in the society and the resolution of the social, educational and economic problems caused by the disease are related to the domain of improvement or outcomes.

### Limitations

The use of only Google Trends data in the research is an important limitation. This means that only the search behavior of people using the Google search engine can be analyzed. This data can be supplemented with trend topics of famous social media applications such as Twitter. In addition, we are not aware of the search data and algorithms used by Google, and we can state that we trust the accuracy of the data. In the analyzes, a regional and temporal analysis was not conducted on the data. In the design of the developed framework, an indirect explanation was made by not directly including the information regarding each level of Maslow's theory of needs (applications and outcomes).

## Conclusion

It was determined that search queries were made for superficial, racist, definitive and non-infodemic attitudes in Turkish society. A systematized COVID-19 development process framework was modeled in order to conceptualize COVID-19 internet searches and to reveal the development processes and outcomes. The framework built on public health epochs illustrates the impact of the conditions created by the pandemic on human needs and the handling of this situation as a disaster. The broad perspective of the framework provides decision makers and researchers with a fundamental area for research, intervention, monitoring and policy. The framework also has the potential to develop interventions to identify the current status of populations affected by the pandemic and to provide efficiency in the use of resources.

## Data availability statement

The raw data supporting the conclusions of this article will be made available by the authors, without undue reservation.

## Author contributions

CÇ and GÖ contributed to the study design, execution, coded transcripts, data collection, and conducted the analysis. All authors helped in data interpretation and contributed to and approved the final manuscript.

## Conflict of interest

The authors declare that the research was conducted in the absence of any commercial or financial relationships that could be construed as a potential conflict of interest.

## Publisher's note

All claims expressed in this article are solely those of the authors and do not necessarily represent those of their affiliated organizations, or those of the publisher, the editors and the reviewers. Any product that may be evaluated in this article, or claim that may be made by its manufacturer, is not guaranteed or endorsed by the publisher.
